# Ground Level Deployment of Wireless Sensor Networks: Experiments, Evaluation and Engineering Insight

**DOI:** 10.3390/s19153358

**Published:** 2019-07-31

**Authors:** Rodrigue Domga Komguem, Razvan Stanica, Maurice Tchuente, Fabrice Valois

**Affiliations:** 1 INSA Lyon, Université de Lyon, Inria, CITI, F-69621 Villeurbanne, France; 2 Faculté des Sciences, Université de Yaoundé I, CETIC, LIRIMA, Yaoundé BP 812, Cameroun; 3 Sorbonne Université, IRD, UMMISCO, F-93143 Bondy, France

**Keywords:** wireless sensor networks, ground deployment, experimental evaluation, links properties

## Abstract

In this paper, we are interested in characterizing the link properties of a wireless sensor network with nodes deployed at ground level. Such a deployment is fairly common in practice, e.g., when monitoring the vehicular traffic on a road segment or the status of infrastructures such as bridges, tunnels or dams. However, the behavior of off-the-shelf wireless sensor nodes in these settings is not yet completely understood. Through a thorough experimentation campaign, we evaluated not only the impact of the ground proximity on the wireless links, but also the impact of some parameters such as the packet payload, the communication channel frequency and the topography of the deployment area. Our results show that a ground-level deployment has a significant negative impact on the link quality, while parameters such as the packet size produce unexpected consequences. This allows us to parameter classical theoretical models in order to fit a ground-level deployment scenario. Finally, based on the lessons learned in our field tests, we discuss some considerations that must be taken into account during the design of communication protocols and before the sensor deployment in order to improve network performance.

## 1. Introduction

Sensors are slowly becoming an integral part of our life, monitoring our heartbeat [1], counting our steps [2], customizing our habitat according to our preferences [3], or detecting the presence of intruders in areas we wish to protect [4]. For ease of deployment and economic reasons, most of these sensors are integrated into wireless devices which transmit the collected information through wireless sensor networks (WSN) to a central server. As a consequence, multiple WSN platforms appeared on the market (e.g., Waspmote [5], NI WSN [6], NODE+ [7], Zolertia Z1 [8], etc.), providing generic hardware and software on the networking side, and a variety of interchangeable physical sensors, fitting numerous use cases.

Several applications require sensors to be deployed at the ground level: vehicular traffic monitoring on a road segment [9], bridge and tunnel monitoring [10], or dam monitoring [11] are some examples among others. As we show in the following, a node deployment at ground level has a significant negative impact on the properties of the wireless links that form the WSN. Our main goal in this study was to assess whether off-the-shelf WSN platforms, not designed specifically for this use case, can be used in a ground-level deployment. To reach this goal, we answerED many questions: (i) What are the key differences between a road surface radio link and a radio link with nodes deployed at a given height? (ii) What are the radio link properties between two nodes deployed at the ground level? (iii) What is the impact (in terms of packet reception ratio (PRR) or received signal strength indicator (RSSI)) of parameters such as transmission power, packet size or communication channel frequency? (iv) What is the impact of the topography of the deployment environment on the link properties?

The main particularity of WSNs is their limited resources: these networks are constrained in energy, storage, processing and communication capability. Energy is the strongest constraint because nodes are usually equipped with batteries of limited capacity [12,13]. In addition to the energy constraint, because of the environmental constraints (obstacles, variable meteorological conditions, etc.), the instability of the radio link is another factor which significantly influences the performance of these networks [14,15]. Topology construction and maintenance, localization, data aggregation and routing are some key operations in WSN which need to exploit reliable wireless links. Link quality has an important impact on these upper-layer protocols. Then, understanding the link properties in order to integrate them in the design of communication protocols is a real challenge in WSN. Several works in which the authors theoretically model the radio channel [16,17,18] or experimentally evaluate the impact of the deployment environment [14,15] on the link quality/properties are proposed in the literature.

However, to our knowledge, none of the previous experimental studies consider the case where sensors are deployed at the ground level. Our main motivation in this paper is to highlight the significant differences that exist between a wireless link at the ground level and one situated at height. Considering sensors deployed at ground level, we also investigate in this paper the impact of some parameters such as the packet size, the communication frequency and the topography of the area. To understand the properties of radio links in a WSN with nodes deployed in such conditions, we conducted an extensive experimental campaign in which we evaluated the impact of different parameters. Our contributions can be summarized as follows:

- We experimentally show a significant difference between a ground surface deployment WSN and a WSN in which nodes are deployed at a given height.
- We measure the impact of several communication parameters on the radio link properties in a WSN with sensors deployed at ground level.
- We compare the obtained results with classical theoretical models, showing that a careful parameterization of the models gives accurate results in general, but some particular situations (very small payload and uphill deployment) deviate from these theoretical explanations.
- We propose a set of guidelines that, if followed, allow using off-the-shelf WSN platforms for ground-level deployment.

The remainder of this paper is organized as follows. In Section 2, we present the literature results concerning the analysis of link properties in WSN. Theoretical radio channel models and their application in a ground-level deployment are discussed in Section 3. In Section 4, we present our experiment methodology and setup, and we also describe the platform used. Section 5 contains the results of our experimental evaluation and a thorough analysis of the impact of different parameters on the link quality. Finally, Section 7 summarizes our recommendations for WSN deployment and protocols design, before concluding in Section 8.

## 2. Literature Review

Numerous studies on the evaluation of radio links in WSN are proposed in the literature. Some authors propose theoretical models [16,17,18] of the wireless channel, while others adopt experimental approaches [19].

In [18], the authors proposed a coexistence model of two standards (IEEE 802.15.4 and IEEE 802.11b/g), which exposes the interactions between these technologies. They analyzed the vulnerabilities low power IEEE 802.15.4 technology face compared to technologies such as 802.11b/g, which use a higher transmission power and work in the same frequency band. Their results show that, when IEEE 802.11b/g interference occurs, the throughput of IEEE 802.15.4 decreases drastically. Our experimental results presented below also highlight that the link quality in IEEE 802.15.4 standard is influenced by interference from other technologies.

Experimental studies have demonstrated that there is a large transitional region (a geographical area) in the communication area of a wireless node, characterized by a significant level of unreliability and asymmetry. Therefore, in [16,17], the authors derived the analytical expression of the packet reception rate distribution as a function of distance, as well as the location and extent of the transitional region. Their results show that the environment (path-loss exponent snf noise floor) and the hardware (hardware variance and output power) have an impact on the size of the transitional region. In this study, we used a single (and homogeneous) hardware platform. Nevertheless, our results show that, from one deployment area to another, the link quality can drastically change, due to the local environment and the precise node location.

Because of the complexity of wireless sensor networks, the designers of communication protocols generally use simplifying assumptions when building their protocols. Thus, in most medium access control (MAC) or routing protocols, a common approach is to assume that the area covered by a signal emitted by a sensor $S_{i}$ is a circle of radius *R*, where *R* is the maximum communication range of node $S_{i}$. This implies that a message transmitted by $S_{i}$ is received by all nodes located at a distance less than or equal to *R* from $S_{i}$. This implicitly assumes an isotropic communication area and symmetrical link between a couple of nodes. However, it is established that such assumptions are far from the reality [20]. Our experimental results show that asymmetric links are even more common when nodes are deployed at the ground level.

In a comprehensive experimental study [14], the authors measured the quality of the radio link in two indoor and one outdoor environments. They tested the validity of several assumptions usually made in conceptual models used to theoretically evaluate communication protocols. These include the *stability* assumption (i.e., the link quality in terms of packet reception ratio changes slowly compared to data rate), the *channel* assumption (i.e., the link quality is the same on all frequency channels), the *spatial* assumption (i.e., losses on different links are independent) and the *acknowledgment* assumption (i.e., acknowledgment and packet delivery ratios are the same). Their results show that none of these assumptions holds.

Focusing on outdoor deployments [15], the authors evaluated the link quality in three different environments: two tunnels (one with vehicular traffic and another without vehicular traffic) and a vineyard. Their study shows that the link quality in the tunnels is significantly different from other classical WSNs. Indeed, the authors observed that, in tunnels, links are generally stable and long-range compared to the vineyard deployment, with the tunnel behaving as a waveguide. While the experiments described in [14,15] do not consider the scenario of ground-level deployment, an important conclusion, which we observed in our experiments as well, is the high dependence of the link quality on the deployment environment.

Moreover, not only spatial properties of link have been observed, but also seasonal properties [21,22,23]. In [22,23], the authors evaluated the impact of the weather on the link quality in a WSN. Their results show a daily and seasonal fluctuation of link quality. In [23], the authors measured over a period of six months the effects of weather on IEEE 802.15.4 links. By measuring the correlation between the variations in the packet reception ratio (PRR) and the signal strength indicator (RSSI) with four selected meteorological factors (temperature, absolute humidity, precipitation and sunlight), the authors showed that the PRR and the RSSI correlate the most with the temperature (better link quality when the temperature is lower). In [22], the authors designed a low-cost experimental infrastructure to vary the on-board temperature of sensor nodes and their results also show a correlation between link quality and temperature. In [21], the authors proposed a new platform and used it to estimate the parameters of an over-water radio link, through two experiments: one conducted in the morning and another in the afternoon. From the morning to the afternoon experiment, the temperature and the relative humidity reduced by about 2 ${}^{{}^{\circ}}$C and 25% respectively. Their results shown an increasing of the radio range from 40 m in the morning to 70 m in the afternoon. The results presented in [21,22,23] show a poor link quality in presence of high temperature. In our case, since temperature is higher at ground level, this brings a negative impact on link quality compared to links placed higher above ground.

Experimenting at large scale requires a lot of hardware and is a fastidious and time-consuming task. Therefore, several testbed platforms have been deployed all around the world to allow faster experimentations, with various sizes, hardware, topologies, and degrees of flexibility [24,25,26,27]. These testbeds are usually deployed in indoor environments, but allow conducting complex and easily reproducible experiments. In these testbeds, a user usually selects and configures the set and type of sensor it wants for its experiment. For some testbeds, it might be possible to select sensors in order to guarantee the main radio link property considered in this paper: radio link at ground level. However, in our work, we preferred conducting experiments in a real environment, which allowed us to evaluate, for example, the impact of the topography.

To summarize, previous results show that link properties in WSN usually depend on hardware properties, radio communication parameters, meteorological conditions and deployment environment. This should be taken into account when designing communication protocols or mechanisms for WSN. In most experimental setups, the deployment choices usually depend on the desired objectives and the platform used: sensors are usually attached on some support, the nodes may be configured with an internal or external antenna. In our study, we considered an off-the-shelf WSN platform [28], with nodes configured with internal antennas and deployed outdoor on the ground surface. Our goal was to characterize the wireless links in such settings, taking into account radio parameters such as the packet size or the communication channel frequency, as well as environment properties such as its topography. Another parameter which may have an impact on the radio link properties, but not investigated in this study, is the transmission power [29].

## 3. Communication at Ground Level: Radio Channel Modeling

As discussed above, several applications require sensors to be deployed at ground level. However, the existing WSN platforms were never evaluated in such conditions.

Different metrics can be used to measure the quality of a communication link. Taking a networking point of view, we adopted the packet reception ratio (PRR) as the main metric in our study, as this allows a straightforward derivation of application layer metrics; however, we also provide extensive Received Signal Strength Indicator (RSSI) results in the following, as a complementary metric. As well established metrics, the PRR and RSSI can also be computed theoretically, using standard communication models. Therefore, before detailing our experimental results, we use a similar reasoning to the authors of [16,17] and we derive analytically the PRR and RSSI, to allow a fair comparison between experimental and theoretical results in Section 6.

### 3.1. Received Power

When an electromagnetic signal propagates, its strength decreases not only because of the distance between the transmitter and the receiver, but also because of other physical phenomena, such as diffraction, reflection or scattering [30]. The signal attenuation, or path-loss, is related to the environment characteristics. Its value depends on the distance between the transmitter and the receiver. It also depends on the position, the shape and the dielectric properties of objects on the signal path. One of the most common radio propagation models, widely used in WSN, is the log-normal path-loss model [17,30] which is given by Equation (1):

(1) $$PL\left(d\right)=PL\left(d_{0}\right)+10\alpha log_{10}\left(\frac{d}{d_{0}}\right)+X_{\sigma }$$

In Equation (1), PL(d) represents the path-lost at a distance *d* between the transmitter and the receiver. On the right side of Equation (1), the first two terms ($PL\left(d_{0}\right)+10\alpha log_{10}\left(\frac{d}{d_{0}}\right)$) represent the signal path loss produced by the distance *d* between the transmitter and the receiver (ideal path-loss), while $X_{\sigma }$ is a Gaussian random variable with mean 0 and variance σ (standard deviation due to multipath effect), which represents the path loss due to shadowing effects. The parameters α (also known as the path loss exponent) and σ depend on the propagation environment and they are generally obtained from field tests in different scenarios. However, from our knowledge, suitable values have never been determined for these parameters in a ground-level deployment scenario. Instead, for propagation that approximately follows a free-space or two-ray model, α can be set to 2 or 4, respectively [30]. Furthermore, in [30,31], the authors proposed an approach to calculate the value of α in a given environment, given measured data. This approach is used to estimate the values of α and σ in Section 6.

The parameter $PL(d_{0})$ is the path loss at a reference distance (distance at which the communication area can be assumed to be a perfect disc) $d_{0}$; its value can be obtained empirically or analytically using the free-space propagation model at distance $d_{0}$. Using an analytical approach, $PL\left(d_{0}\right)=20log_{10}\left(\frac{4\pi d_{0}}{\lambda }\right)$, where λ is the wave-length of the transmitted signal. Finally, the received power $P_{r}\left(d\right)$ at a distance *d* from the transmitter is given by Equation (2):

(2) $$P_{r}\left(d\right)=P_{t}-PL\left(d\right)$$

**Figure 1:**
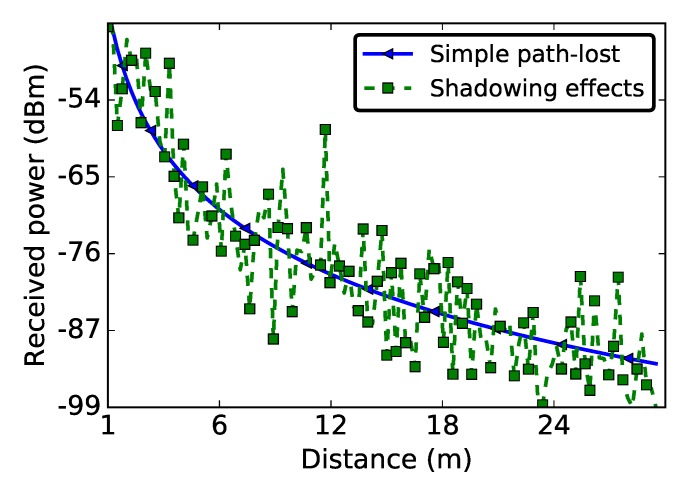
Theoretical received signal strength over a WSN link: α=3.56, σ=6.06, $P_{t}$ = 0 dBm, $d_{0}$ = 1 m.

Figure 1 shows theoretical values of the received power $P_{r}$ (see Equation (2)) as a function of distance, using a transmission power of 0 dBm and a reference distance $d_{0}$ of 1 m. $PL(d_{0})$ is evaluated using the free space propagation model [30] and is equal to 40.33 dB. The values of α and σ (3.56 and 6.06, respectively) are computed from our empirical data, as discussed in Section 6. To demonstrate the impact of the multi-path fading effect, this figure also depicts the received power in an ideal scenario, with no multi-path fading (i.e., $X_{\sigma }=0$ in Equation (1)). In this simple ideal channel, the path-loss is a logarithmic function of the distance between the transmitter and the receiver.

### 3.2. Packet Reception Ratio

The probability of successfully receiving a packet over a wireless link depends on the packet length and the probability of successfully receiving each bit of the packet. The number of bits transmitted at the physical layer depends on the encoding scheme. The probability of successfully receiving a bit, or its complementary, the bit error rate (BER), depends on the used modulation and on the signal-to-noise ratio (SNR) at the receiver side.

For a received power $P_{r}\left(d\right)$ at distance *d*, the SNR $\gamma_{d}$ is given by Equation (3):

(3) $$\gamma_{d}=\frac{P_{r}\left(d\right)}{P_{n}}$$

where $P_{n}$ is the noise floor (the total sum of noise in area and frequency range). In interference-free environments, $P_{n}$ is given only by the thermal noise and it is constant. Nevertheless, in most environments, $P_{n}$ varies with time, either because of interference or because of changes in temperature. Similar to the work in [32], we consider this realistic hypothesis (variable $P_{n}$) and model $P_{n}$ as a Gaussian random process of mean 0 and standard deviation $X_{\gamma }$.

For a quadrature phase-shift keying (QPSK) modulation, such as the one used by platforms used in our experiments, described in Section 4, the BER at distance *d*, $P_{e}^{d}$ is given by Equation (4) [17]:

(4) $$P_{e}^{d}=\frac{1}{2}erfc\left(\sqrt{\gamma_{d}\frac{B_{N}}{R}}\right)$$

where *R* is the bit data rate, $B_{N}$ is the bandwidth of the additive white Gaussian noise, and erfc(x) is the complementary error function, defined as $ercf\left(x\right)=\frac{2}{\sqrt{\pi }}\int_{x}^{\infty }\;e^{-t^{2}}\;dt$.

Then, the probability $p_{d}$ of successfully receiving a packet of size *f* bits at distance *d* is given in Equation (5):

(5) $$p_{d}≃{\left(1-P_{e}^{d}\right)}^{f}$$

For several encoding schemes and modulations, the BER and the PRR are given in [17].

**Figure 2:**
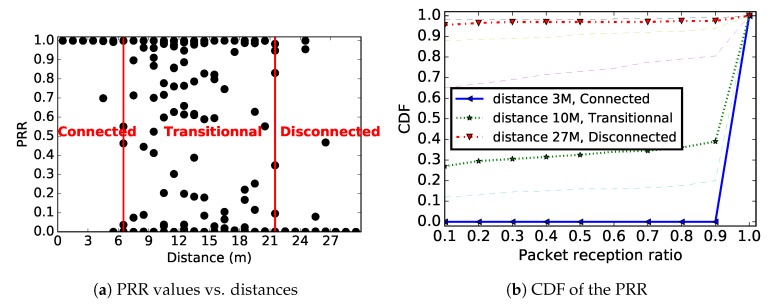
Theoretical packet reception ratio over a WSN link: non-coherent QPSK modulation, NRZ encoding, packet size 100 Bytes, α=3.56, σ=6.06, and $P_{t}$ = 0 dBm.

Figure 2a shows the theoretical PRR values for links of different distances. For the log-normal shadowing model, we used the same parameters values used for Figure 1. We considered a QPSK modulation, NRZ encoding and a packet sized of 100 Bytes. For each distance, we generated many PRR values. As in [16,17], we distinguish three different areas in this figure: a *connected area*, in which links are almost perfect (PRR≈1); a *transitional area*, in which the links are unstable; and a *disconnected area*, in which we have very poor or no links. Figure 2b shows the cumulative distribution function (CDF) of link PRR for several distances. It highlights the distances of 3 m, 10 m and 27 m, located in connected, transitional and not connected areas, respectively. The results presented in Figure 2b show that most of the links in the network are expected to be either of poor quality (PRR less than 10%) or of good quality (PRR superior to 90%), with very few links in the transitional category.

## 4. Experiment Overview

To understand whether off-the-shelf hardware [28] behaves as predicted by theoretical models, we conducted an extensive evaluation campaign. This section discusses the deployment scenarios, the experimentation material and methodology, as well as the selected evaluation metrics.

### 4.1. Sensors Characteristics and Deployment

For all experiments discussed in this paper, we used the TelosB platform [28]. This platform is equipped with a 8 MHz microprocessor, a 10 KB RAM, a 1 MB external flash memory and a set of sensors (temperature, humidity and light). In all our experiments, the collected data were stored in the external memory.

For communication, TelosB uses the CC2420 radio module. This module is IEEE 802.15.4 compatible and it is widely used for industry and research in the field of low power wireless sensor networks. The CC2420 radio shares with other technologies, e.g., IEEE 802.11 (WiFi) and Bluetooth, the unprotected ISM frequency band (2400–2483.5 MHz). In other words, if a WSN were deployed in an area covered by equipments using these different technologies, the communication of nodes in the network would interfere with these other devices. Following the IEEE 802.15.4 protocol [33], the spectrum is divided into 16 channels of 2 MHz bandwidth and 5 MHz spacing between consecutive channels. The CC2420 module also offers 32 different output power levels, ranging from −25 dBm to 0 dBm. The transmission power and the communication channel can be programmed by changing the value of particular CC2420 registers.

**Figure 3:**
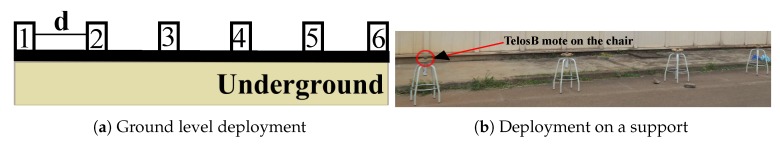
Sensors deployment.

In our experiments, a set of sensors was linearly deployed with a fixed distance *d* between two consecutive sensors. In each experiment, the same communication protocol, avoiding in-network interferences, was executed by all sensors. To assess the impact of ground-level deployment on the quality of wireless links, we also studied a scenario where nodes follow a similar linear deployment, but placed on a support, tens of centimeters above ground. We did not control the human movement in the deployment area or the external radio interference. Figure 3 shows the sensors deployment in the two context: ground level and on a support.

### 4.2. Communication Protocol

In all our experiments, each sensor executed the same protocol, which we implemented in Contiki OS 2.7 [34]. For a deployment using *N* sensors, all nodes were initialized with identifiers ranging from 1 to *N*. To avoid internal interferences, nodes communicated using a round-robin algorithm. When it was a node’s turn to send messages, it broadcasted *M* messages at a frequency of one message per 500 ms. This upper layer synchronization allowed us to disable the carrier sense mechanism and practically use a transparent MAC protocol.

The transmitted messages contain the identifier of the sender, a sequence number, the transmission power used by the message sender and a dummy application payload. Upon message reception, a node stores in its external flash memory all the information found in the message, as well as information on the RSSI and the link quality indicator (LQI) provided by the receiver radio module. This communication protocol is described by Algorithms 1–3. Algorithm 1 was executed by each sensor at initialization, while Algorithms 2 and 3 were callbacks called for messages transmission and reception respectively.

**Algorithm 1** Sensor initialization—called at sensor initialization.**Input:***N*: Number of sensors, *F*: Communication frequency, *P*: Transmission power, φ: Transmission
  period, *i*: Sensor rank
1:ConfigureCommunicationFrequency(F)   2:ConfigureTransmissionPower(P)   3:TxStartDate← GetTxStartDate(*i*, *N*, φ)   4:SetTimer(TxStartDate, TxCallBack)


**Algorithm 2** TxCallBack—called when a sensor wants to send messages.**Input:**PayLoadSizeList: The list of different payload sizes, *P*: Transmission power
1:sequenceNumber ← 0   2:**for** plsize in PayLoadSizeList **do**3:  SendBroadCastMessage(plsize, sequenceNumber, P)   4:  SetTimer(Now+φ, TxCallBack)   5:sequenceNumber ← sequenceNumber + 1   6:**end for**

**Algorithm 3** RxCallBack—called each time a message is received.**Input:**RcvMessage: The new message received   
1:RSSI ← GetRSSI(RcvMessage)   2:LQI ← GetLQI(RcvMessage)   3:SenderID ← GetSender(RcvMessage)   4:sequenceNumber ← GetSequenceNumber(RcvMessage)   5:PayLoadSize ← GetPayLoadSize(RcvMessage)   6:P ← GetTransmissionPower(RcvMessage)   7:SaveDataToExternalMemory(RSSI, LQI, SenderID, sequenceNumber, PayLoadSize, P)


### 4.3. Evaluation Criteria

As explained, for each received message, the CC2420 module allowed logging the RSSI and LQI values. The RSSI is an estimation of the signal power at the receiver. In the TelosB data-sheet [28], the received signal power in dBm, $P_{r}$, is defined by:

(6) $$P_{r}=RSSI\_VAL-RSSI\_OFFSET$$

where RSSI_OFFSET, empirically found in the data-sheet [28], is equal to −45 dBm.

In addition to the RSSI, the LQI is also a widely used metric to characterize the quality of the radio link in low power wireless sensor networks. However, in this study, we preferred to use the PRR instead of LQI as a link quality metric, as the PRR is more meaningful at the network layer, and it is calculated over a longer time interval (i.e., several message receptions). Practically, we used the sequence number of the received messages to calculate the PRR value over batches of 20 messages.

**Figure 4:**
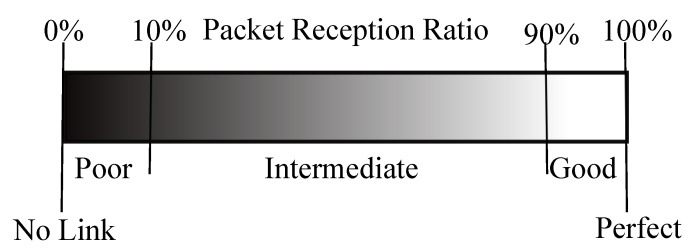
Link definition.

Figure 4 shows how the link is defined as a function of the measured PRR, following guidelines similar to those in [14]: if no packet is received between two nodes, there is *No Link* between this pair of nodes. A link on which less than 10% of packets are received is considered as *Poor*. If 10–90% of packets are received, the link is considered as *Intermediate*. A link on which more than 90% of packets are received is considered as *Good*, and when 100% of packets are received the link is considered *Perfect*.

Another important property is the link symmetry: between two nodes A and B, we have the oriented link A → B that materializes the messages transmitted by A, and B → A for messages where B is the sender. Depending on the quality of links A → B and B → A, we have a symmetrical (the two links exist) or asymmetrical (only one of the two links exists, either A → B or B → A) link between nodes A and B. To measure the asymmetry of a link, we used both the PRR and RSSI, by designing two metrics, $asm_{AB}^{PRR}$ and $asm_{AB}^{RSSI}$, as follows:

(7) $$asm_{AB}^{PRR}=\left(|PRR_{AB}-PRR_{BA}|\right)$$

where $PRR_{AB}$ is the PRR measured from A to B, and

(8) $$asm_{AB}^{RSSI}=\left(|{\bar{RSSI}}_{AB}-{\bar{RSSI}}_{BA}|\right)$$

where ${\bar{RSSI}}_{AB}$ is the mean RSSI measured for messages transmitted from A to B for each PRR measured by B. Please note that the latter metric is only meaningful if at least one message is received in both directions. Otherwise, the link is considered unidirectional. Finally, the percentage of unidirectional links, $U_{l}$, is also an indicator we used to measure the symmetry of the links in the network.

### 4.4. Experiment Methodology

Our methodology, for each experiment, can be summarized by the following steps:

1. A set of *N* TelosB sensors was used, with all the nodes configured to use one of the 16 communication channel defined by the IEEE 802.15.4 standard.
2. The sensors were linearly deployed, at ground level or on a support at a height of 57 cm above ground, with a fixed distance *d* between two consecutive nodes.
3. To evaluate the impact of the message size, each node sent *M* messages for each application payload from 2 bytes to 100 bytes.
4. All messages were sent in broadcast mode, at a frequency of two messages per second.
5. All nodes used the communication protocol described in Section 4.2.
6. When a node received a message, it stored in its external flash memory the message sequence number and the RSSI. The message sequence number was used to calculate the PRR.
7. The experiment ended when every node in the deployment had sent all its messages.

In the following sections, we give, for each experiment we discuss, the number of sensors *N*, the number of messages *M* and the distance *d* between two consecutive nodes.

## 5. Experimental Results

Having discussed the experiment set-up, our goal in this section is to characterize the radio links in a WSN with nodes deployed on the ground surface, taking into account some parameters such as the deployment environment, the communication channel frequency and the packet size. We start by comparing the wireless link properties at ground level and in an above-ground deployment, followed by an analysis of the different parameters with an impact on link quality, and we finish by integrating these experimental results with theoretical values obtained using the model described in Section 3.

### 5.1. Comparative Study of Ground-Level and Above-Ground Deployment

As noted in Section 2, to our knowledge, none of the experimental results presented in the literature consider the case where nodes are deployed at ground level. For this evaluation, we conducted two sets of experiments. In the first set of experiments, six sensors were linearly deployed on the ground with a fixed distance of 3 m between them. In the second set of experiments, sensors were linearly deployed but, in this case, each sensor was deployed on a support of height 57 cm. In both experiments, channel 26 of the IEEE 802.15.4 standard was used by sensors for communication. However, note that Section 5.3 presents in detail the evaluation of the impact of the communication channel in a WSN with nodes deployed on the ground surface. Nodes were configured to use a transmission power of 0 dBm and an application payload of 82 bytes was added as user data for each packet transmitted in the network. As discussed in Section 5.2, we further evaluated the impact of the packet size on the link quality.

**Figure 5:**
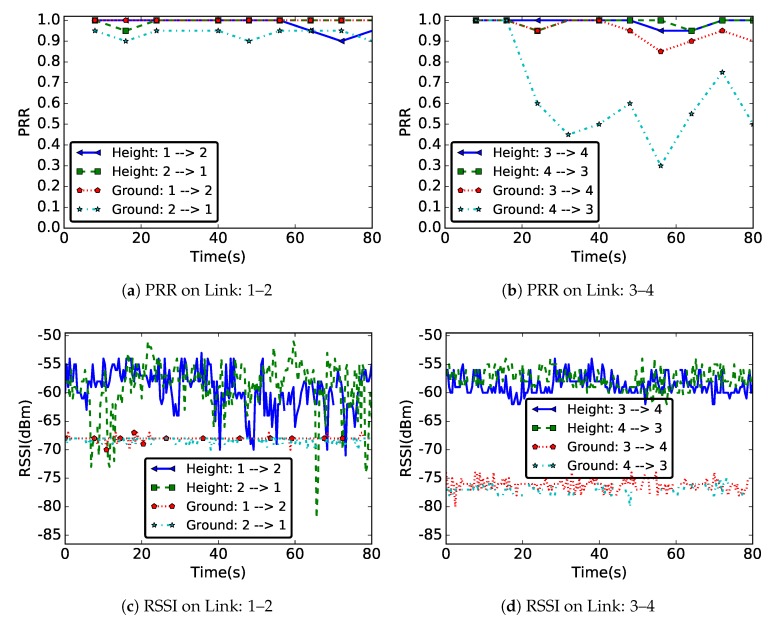
Variation of PRR and RSSI values over time on wireless links 1–2 and 3–4, with the same distance of 3 m, in two different deployments: one with sensors deployed at ground level (labeled “Ground”) and another with sensors deployed on a support of height 57 cm (labeled “Height”).

Figure 5 presents the PRR and RSSI values on forward and backward links of pairs of nodes (1,2) and (3,4) as a function of time, when nodes were deployed at ground level and at height. From node 1’s point of view, related to node 2, the forward link is the link 1→2 and the backward link is the link 1←2. We considered nodes located at different position in the network, but with the same separation distance of 3 m. This allowed analyzing and comparing not only the temporal properties of the links in the two deployments, but also some spatial properties.

#### 5.1.1. PRR and RSSI Values

As mentioned in Section 4, we considered the RSSI and PRR values to characterize the radio communication links. These metrics can be influenced not only by hardware properties, but also by deployment area characteristics. The values of PRR and RSSI presented in Figure 5 show that, in our experiments, the links were most affected when nodes were deployed at ground level. Indeed, the values of measured RSSI or PRR at ground level were always smaller compared to the values measured for a deployment at height. This is particularly true in the case of RSSI, where the difference could reach 10–20 dBm. Since the same hardware was used for both deployments, this significant difference in the value of RSSI and PRR measured in the two experiments was mainly due to the environment properties. These properties were time and link-position dependent. In the following, we compare, for the two deployments, the temporal and spatial properties.

An important observation can be made in Figure 5: the two metrics, RSSI and PRR, have different behaviors. While the difference in terms of RSSI between ground-level and aboveground scenarios is always significant, this is not the case for PRR, meaning that a similar PRR can be obtained at highly different RSSI levels. Moreover, the opposite is true as well and, at similar RSSI levels, the PRR can be quite different, as shown for the two directions of link 3–4. The results presented in this section show that the PRR is the best metric to use at network layer to select the link which guarantees the relevant metric transmission reliability.

#### 5.1.2. Temporal Properties

As observed, the RSSI or the PRR values were better at height compared to a ground-level deployment. Figure 5 also shows that the temporal properties of links were different in the two deployments, with an opposite behavior when considering PRR and RSSI values. At ground level, while we had a stable radio link, but poor quality, in terms of RSSI values, we observed a relatively high fluctuation of the measured PRR values. A different behavior was noticed for the deployment at height, where we had stable PRR values and a high fluctuation of RSSI values.

We also note that the relatively high RSSI values obtained at height resulted in better PRR values than the stable RSSI values measured at ground level. As hinted by Figure 5b and further discussed in Section 5.1.4, the ground-level deployment also resulted in a significant number of unidirectional links. Indeed, despite very similar RSSI values, link 3–4 showed highly different PRR values on the two directions, node 4 receiving many more messages than node 3.

An explanation for the low RSSI values in the ground-level deployment might come from the increased number of signal reflections, hence resulting in multi-paths [30]. As both the transmitter and the receiver were close to the ground, this phenomenon was to be expected. On the other hand, in the case of a deployment at height, the propagation environment was a more open space (compared to a deployment at ground level), but also more sensitive to different sources of reflexions (e.g., a person moving around the deployment area). We hypothesized that the fluctuations of the RSSI values in the case of a deployment at height was due to this property of the environment.

#### 5.1.3. Spatial Properties

In practice, the reliability of many hardware-based localization algorithms [35,36] depends on the correlation of the RSSI with the distance. However, this correlation is questioned by our experimental results, as well as by other previous field tests [20]. For example, links 1–2 and 3–4 presented in Figure 5 covered the same distance (3 m), but they were located at different positions. However, as Figure 5 highlights, the properties of these two links were different, especially in the case of the ground-level deployment. Indeed, the shape of the road around each node might be different from one sensor to another, which leads to different link properties at different points in the network. We argue that such spatial considerations, as well as temporal variations, must be taken into account by WSN designers.

#### 5.1.4. Link Asymmetry

Link asymmetry is another property that must be taken into account when designing WSN protocols. Looking at the RSSI or PRR measured on forward and backward links of pairs of nodes (1,2) and (3,4), we noticed a significant difference: for a ground surface deployment, Figure 5b shows a much higher PRR on the forward link 3→4 than on the backward link 4→3.

Beyond this illustrative example, Table 1 presents statistics concerning the difference of PRR and RSSI values measured on forward and backward links on all nodes, as well as the number of unidirectional links. These results show that the asymmetry problem is general in WSN, and not restricted to ground-level deployments. Indeed, Table 1 shows that, while the average difference of measured PRR on forward and backward links was higher at ground level, the maximum difference of the measured PRR and the maximum or the average difference of measured RSSI on forward and backward links were even higher for a deployment at height. However, we also note that, while there were no unidirectional links when the sensors were deployed at height, we had 8% unidirectional links in the case of ground-surface deployment. This means that, for a deployment at height, even if there might temporarily exist a significant difference in values measured between two nodes, the forward and backward links are usually available in the two communication directions.

#### 5.1.5. PRR and RSSI Distribution

**Figure 6:**
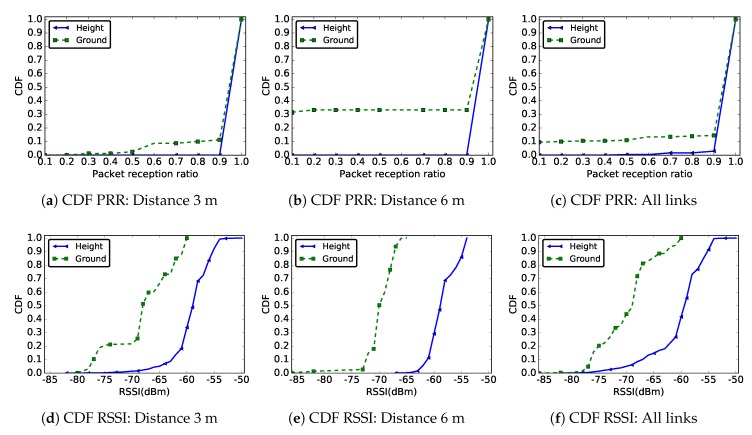
CDF of PRR and RSSI for distances 3 m and 6 m and for all links in the network in two different deployments: sensors deployed at ground level and sensors deployed on a support of height 57 cm.

Figure 6 shows the CDF of the PRR (Figure 6a–c) and RSSI (Figure 6d–f) values measured for the two deployments. These results are shown for radio links of length 3 m, 6 m, and for all existing links in the network. As in the case of Figure 6, these results also confirm the degraded quality of a link in a ground surface deployment compared to a height deployment.

For example, as shown in Figure 6, nearly 80% of the messages were received with a RSSI less than −65 dBm at ground level, while, in a deployment at height, more than 80% of the messages were received with RSSI higher than this value. The differences were even more visible when one considered just links of distance 6 m, where the ranges of the measured RSSI values in the two deployments were actually disjoint.

**Figure 7:**
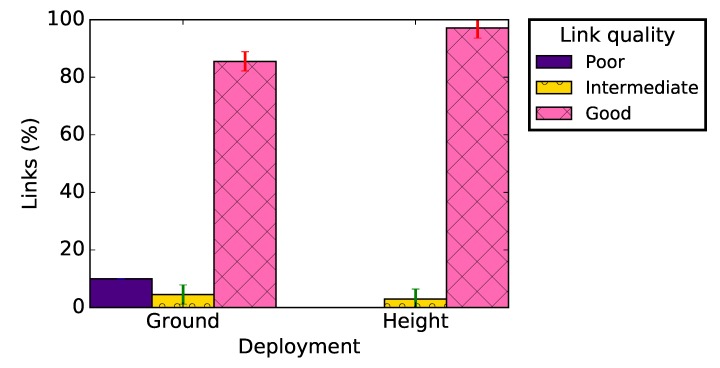
Link quality classification for a deployment at ground level and at height.

Figure 7 gives the distribution of the PRR of radio links in the network according to their quality (see Figure 4) and deployment, taking into account all the network links. Regardless the deployment conditions, these results show that most of the radio links in the network were good with very few intermediate links (see Figure 4). These results are consistent with the theoretical ones presented in [17] and in Section 3, and with other experimental results [14]. We note, however, that almost 10% of links at ground level were poor, while all links in the case of a deployment at height were either intermediate or good (with almost 95% good links).

#### 5.1.6. Discussion

Considering the temporal and spatial properties of radio links, as well as the link asymmetry, our results conclude to a deteriorated radio link quality when nodes are deployed at ground level. The relatively poor quality of wireless links in such deployment is due to the proximity of the ground, which creates multiple signal reflections and can even act as an obstacle to radio wave propagation most of the case. Since reflected and original signal are summed at the receiver, this can produce distortion in the resulting signal and then negatively impact link quality [30].

The temperature at ground level can also be an explanation for this result. Indeed, several studies [21,22,23] show that, the higher is the temperature, the lower is the quality of the link, because of the thermal noise. In our case, with sensors deployed on a road, often in full sunlight, the temperature at ground level was significantly higher than the temperature for the deployment at height.

Although it is well known [14,15,20,21,22,23] that the radio link in low-power WSN is very unstable because of many factors such as the radio properties or the propagation environment, the results presented in this section show that, at the ground level, the radio links quality (measured by the PRR and RSSI values) are more severely affected by such environment. This must be taken into account, not only during the deployment of such networks, but also for designing communication protocols.

On the good side, our results demonstrate that communication between off-the-shelf sensor nodes is possible at ground level. Thus, by using relaying capability of sensors, the door is opened to the numerous applicative use-cases discussed in Section 1. However, results presented in this section only consider one transmission power (0 dBm), one application payload size (82 bytes), one communication channel (number 26), and one deployment area (a relatively flat area). All these parameters may have an impact on the link quality. Therefore, in the following, except the transmission power, which is kept to 0 dBm, we present the analysis of the impact of these parameters on the link properties.

### 5.2. The Impact of Packet Size

The maximum size of an IEEE 802.15.4 frame is 127 bytes. Many experiments were conducted with different packet sizes. Our goal was to evaluate the impact of this parameter on the radio link when nodes ware deployed at ground level. We considered five sensors linearly placed on the ground with a distance of 3 m between two consecutive sensors. The nodes were configured to use channel 26 for communications and 0 dBm as transmission power. We varied the application payload from 2 bytes to 102 bytes.

**Figure 8:**
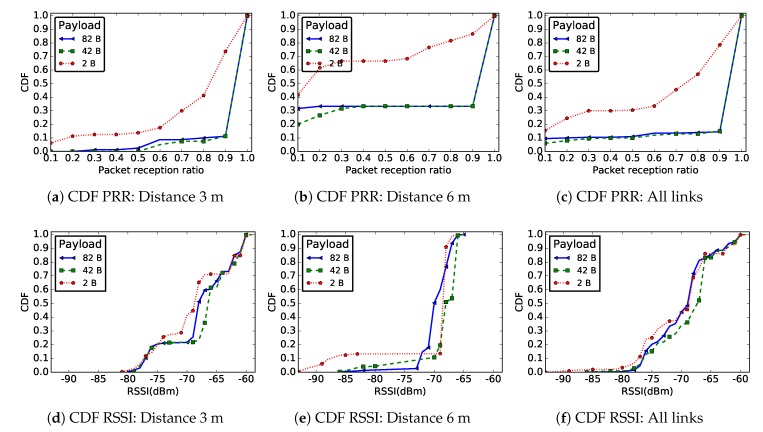
CDF of PRR and RSSI for distances 3 m and 6 m and for all links in the network for different packet sizes.

Figure 8 shows the PRR and RSSI CDF for radio links of length 3 m (Figure 8a,d), 6 m (Figure 8b,e), and for all links in the network (Figure 8c,f). For presentation purpose, only three application payloads are shown in this figure.

**Figure 9:**
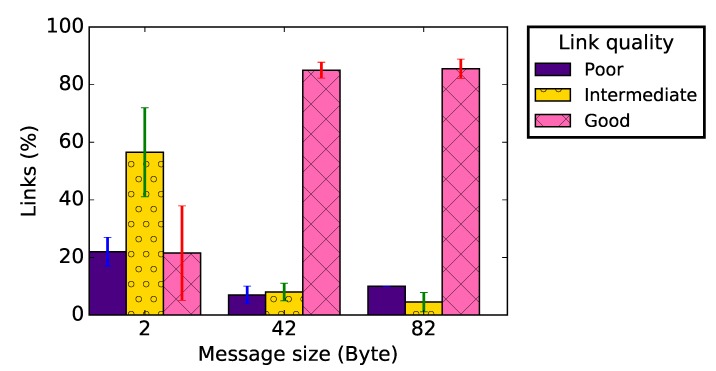
Link quality classification for different packet sizes.

The results do not show a significant difference concerning the RSSI distribution, regardless the application payload. Nevertheless, Figure 8a–c presents a clear difference between the PRR distribution for a very small application payload (2 bytes, i.e. only the sequence number as payload in the packet) and larger ones (42 and 82 bytes). Figure 9 shows that, with an application payload of 2 bytes, there are more intermediate radio links in the network, while for other message sizes more good radio links are present in the network. Similar remarks can be made concerning the radio link asymmetry. Indeed, the data presented in Table 2 show that, when the application payload was 2 bytes, the average difference of the measured RSSI or PRR on forward and backward radio links was the highest. The percentage of unidirectional wireless links was also larger for such a small message size.

We could not totally explain the poor quality of links when the packet payload was only 2 bytes, which might be even due to some operating system bug. However, we can confirm that this phenomenon manifested in all the different settings we tested, and it disappeared for applicative payloads larger than 10 bytes.

### 5.3. The Impact of Communication Channel

Because the ISM band used in IEEE 802.15.4 is shared by other technologies such as IEEE 802.11 and Bluetooth [37,38], interference from other networks influence the link properties in a WSN. To test the impact of the communication frequency on the link quality, we conducted a set of experiments using 5 of the 16 channels (numbered from 11 to 26) defined by the IEEE 802.15.4 standard. For these experiments, four sensors were linearly deployed on the ground surface with a distance of 3 m between two consecutive sensors. Nodes were configured to use a transmission power of 0 dBm. For each channel, experiments were conducted for various application payloads, but results presented in this section consider an application payload of 82 bytes (since, in the previous section, we obtained better links with this application payload).

**Figure 10:**
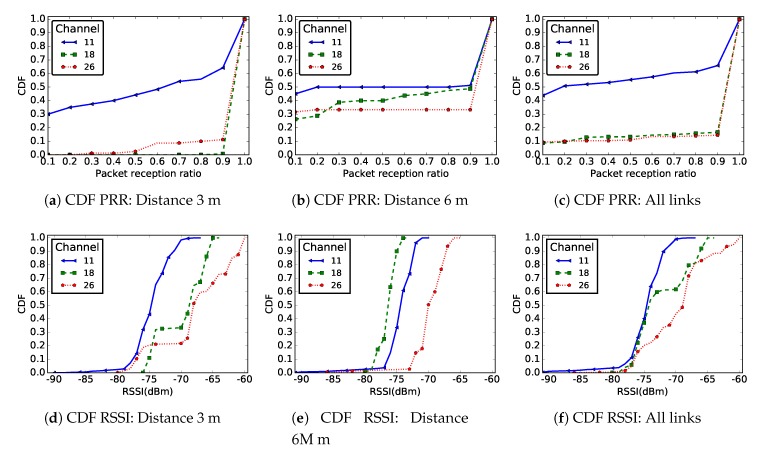
CDF of PRR and RSSI for distances 3 m and 6 m and for all links in the network, for different communication channels.

Figure 10 presents the distribution of the PRR and RSSI on communication channels 11, 18 and 26. In this figure, we consider the cases of links of distance 3 m only and 6 m only, as well as aggregated results for all the links in the network.

**Figure 11:**
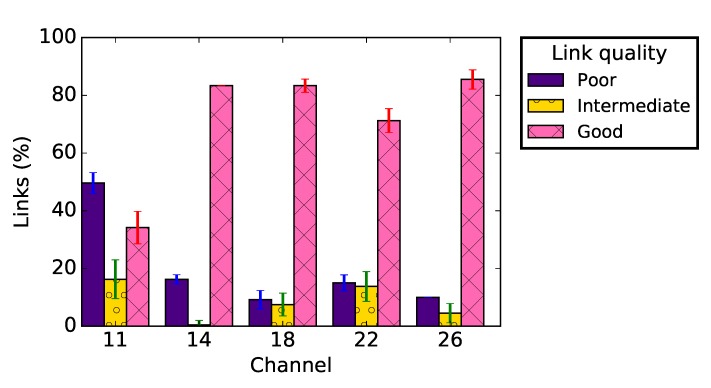
Link quality classification for different communication channels.

These results show that the quality of links was better on channels 18 and 26 compared to channel 11. Figure 11 also shows that, on channel 11, we observed more poor quality links, while more good quality links were observed on other channels. The poor performance of links on channel 11 can also be observed in Table 3, which presents the impact of communication channel on links asymmetry. Even if the PRR measured on forward and backward links were almost equal on channels 11 and 18, the values of measured RSSI were very different in both links. This table shows that almost half of the links in the network were unidirectional when communication channel 11 was used. This lower quality of the links on channel 11 could be related to interference caused by IEEE 802.11 networks deployed on the university campus.

We also observed that the links were better on channel 26 compared to other channels. This was because channel 26 is the only one that does not overlap with the spectrum used by IEEE 802.11 networks [37]. In this section, it is important to note that, for each experiment, all sensors in the network communicated on the same channel. Better performance could be obtained if more advanced MAC layer techniques, such as Time-Slotted Channel Hopping, were used [39,40].

### 5.4. The Correlation between Link Quality and Distance

**Figure 12:**
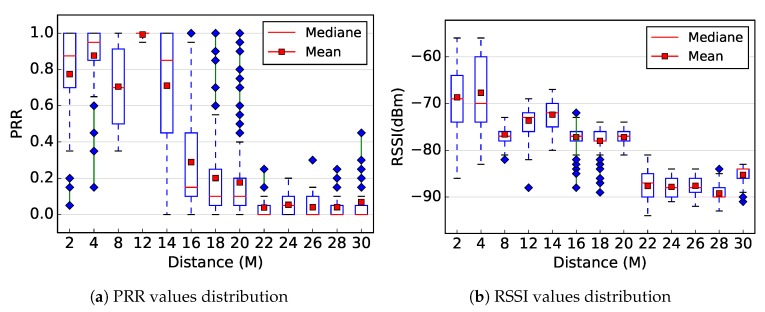
Correlation between the PRR/RSSI and the distance (please not that the *x*-axis is not linear).

To show the correlation between the link quality and the distance separating communicating sensors, we linearly deployed a set of five sensors at ground level. By varying the distance between two consecutive sensors, we performed many experiments. In all experiments, sensors were configured to use channel number 26 and 0 dBm as transmission power. Figure 12 shows the PRR and RSSI as a function of the distance. The red points show the mean PRR (Figure 12a) or RSSI (Figure 12b). The red line in the box represents the median, the bottom edge of the box represents the 25th percentile, and the top edge represents the 75th percentile.

Intuitively, one might think that, the lower is the distance between two sensors, the better is the radio link quality between those sensors. The theoretical model described in Section 3, as well as many others available in the literature [30], implicitly make this theoretical result, particularly for the received power level. In addition, many hardware-free distance estimation solutions proposed in the literature consider this theoretical result [41,42,43]. Simulation tools for WSN also widely use similar models to measure the received power between two sensors.

However, the results presented in Figure 12a for PRR, and particularly those presented in Figure 12b for RSSI, show that such assumptions do not hold in practice. Indeed, Figure 12a shows that the measured PRR was better at 12 m than for shorter distances. This may be due to the road shape or other environment factor. In addition, Figure 12b shows that the RSSI was not a strictly decreasing function of the distance. Therefore, such considerations must be taken into account by protocols designers during theoretical or experimental performance evaluation.

### 5.5. The Impact of Topography

The deployment environment can have an important impact on a radio link. In our case, the shape of the road, networks nearby and the weather were some environment factors that might have influenced the wireless link properties. We therefore performed experiments in another environment in addition to the relatively flat area used thus far. In this second environment, the nodes were deployed on a hill with a steep slope. In this deployment, the sensors were configured to use channel number 26 and 0 dBm as output power for transmissions. The results presented in this section are for an application payload of 82 bytes.

**Figure 13:**
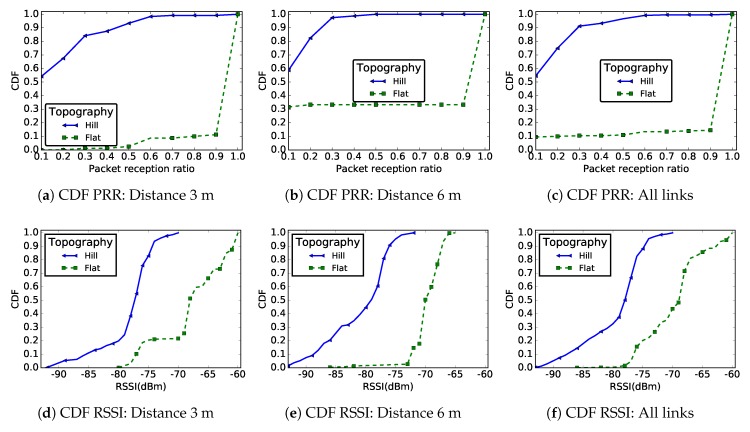
CDF of PRR and RSSI for links with distances 3 m and 6 m, as well as for all the links in the network in the case of a hill and a flat area deployment.

Figure 13 shows the CDF of PRR and RSSI for both deployment areas. Based on these results, we observed links with very poor quality for the hill deployment (labeled “Hill”) compared to links on a flat area (labeled “Flat”), whether we considered the PRR or the RSSI metrics.

**Figure 14:**
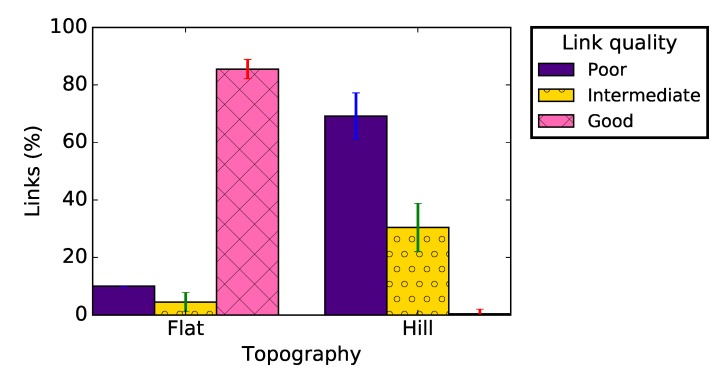
Link quality classification for different deployment environments.

The results presented in Figure 14 complement these findings. Indeed, while we observed 81.5% of good links on a flat area, we observed 70% of poor links on the hill deployment. As in previous sections, the majority of links in the network were either good or bad with few intermediate links. Table 4 presents the links asymmetry parameters for the two different environments: the link asymmetry was also much more pronounced on the hill than on the flat area, with more than 45% unidirectional links. Our tests did not show a significant difference between the PRR and the RSSI values of uphill and downhill links, meaning that the radiation pattern of the CC2420 module antenna was not the reason for these results. We believe that the shape of the deployment environment was the main cause for this poor link quality, resulting in more signal reflections.

## 6. Comparison to Theoretical Results

To strengthen the experimental results presented in this paper, we compared the results obtained in our experiments with those produced by the theoretical model described in Section 3. To compare the empirical and theoretical results, we began by estimating the path-loss exponent (α) and the shadowing standard deviation (σ) from the empirical data [30]. The idea was to find the values of α and σ that minimized the MSE (Mean Square Error) between the simplified model and the empirical power measurements. Using the methodology described in [30], we obtained the values shown in Table 5 in the case of the flat and hill deployments.

We used these values of α and σ as an input for the theoretical model described in Section 3, and then we generated theoretical values for the PRR and RSSI metrics. To check whether the empirical and theoretical distributions are comparable, we used the Mann–Whitney–Wilcoxon (MWW) test [44], a non-parametric test which does not make any assumption on the shape of the distributions. This test allows checking if two distinct sets of data come from the same distribution. The MWW test allowed us to verify the null-hypothesis $H_{0}$: values from the two independent sample sets (i.e., empirical and theoretical data in this case) come from the same distribution.

**Figure 15:**
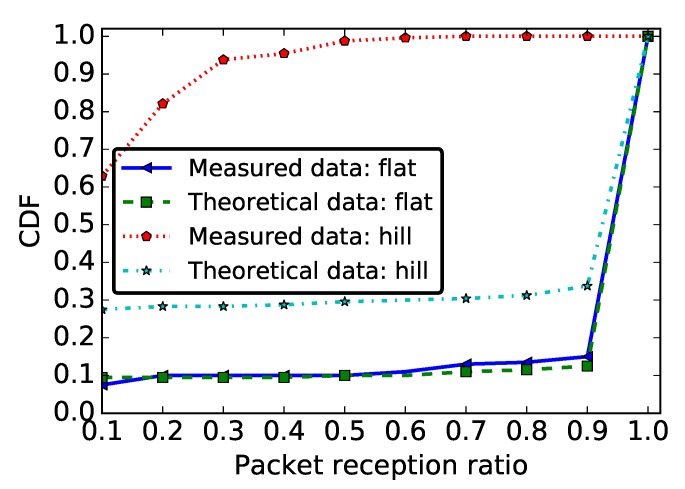
PRR Validation: empirical and theoretical CDF.

In the case of the PRR metric, the MWW test validatef $H_{0}$ in the case of the flat area deployment, but rejected it for the hill deployment. Thus, if we considered a flat environment, the theoretical model was close to the experimentation, while, in the hill environment, there was no correlation between theory and practise. This can also be observed visually in Figure 15, which shows the empirical and theoretical CDF for the flat and the hill deployments. This indicates that the poor radio link quality on the hill deployment was the consequence of a phenomenon that is not taken into account in the theoretical model. We also tested $H_{0}$ for the PRR distribution on every individual link in our deployment: the null-hypothesis was rejected for 15% of links in the flat area deployment and for 37% of the links in the hill deployment.

**Figure 16:**
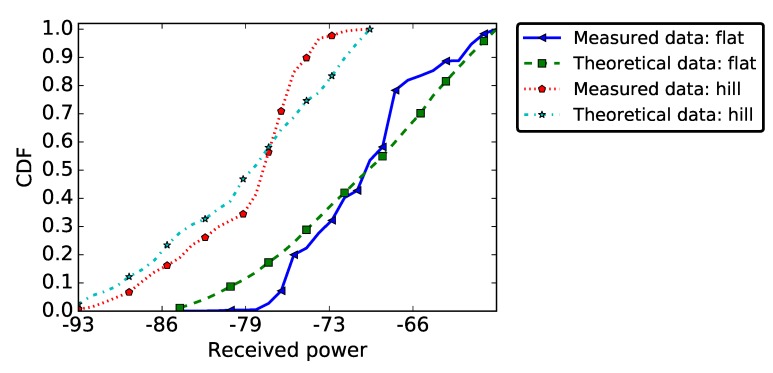
RSSI Validation: Empirical and Theoretical CDF.

Regarding the RSSI metric, the MWW test rejected the null-hypothesis $H_{0}$ for both deployment topographies (flat and hill) (see Figure 16). Thus, there was no correlation between theory and practise results, regardless of the considered environment. This result is in line with the one presented in [45], where the authors conducted multiple statistical tests to check whether the empirical RSSI measured on a link can be approximated by a known probability distribution function. For all the probability distribution functions verified in their study, the null-hypothesis was rejected. This can be explained by the fact that the theoretical model for RSSI considers the received power strength decreases with distance, a property we already showed not to be true in the experiments presented in Section 5.4. In the per-link analysis, $H_{0}$ was rejected for 98% of the links in the flat area deployment and for 68% of the links in the hill deployment.

## 7. Recommendations for WSN Designers

Because WSN at ground level have a lot of application including traffic monitoring, smart parking or bridges monitoring, we believe that engineering recommendations can be drawn from this study. Leading this experimental campaign allowed us to gain a significant insight regarding the deployment of wireless sensor nodes at ground level, as well as regarding the properties of WSN links in general. Based on this experience, we formulate five recommendations for researchers and engineers interested in similar deployments.

Recommendation #1: Account for asymmetric links.

A classical and simple assumption when evaluating the communication protocols in WSN is that the links are symmetric: if node $S_{1}$ hears node $S_{2}$, $S_{2}$ also hears $S_{1}$. However, our results indicate that this property is not always true, especially when nodes are deployed at ground level. Indeed, depending on the settings, up to 40% of the links in the network are unidirectional. This raises several important protocol problems. First, this means that solutions based on acknowledgment (ACK) messages might be inefficient, since the ACKs would be lost despite a successful message reception. Second, routing protocols need to take into account that ingress and egress routes might have very different properties.

Recommendation #2: Remember that the correlation between link quality and distance is weak.

The physics of radio wave propagation predict a monotonic decrease of the received power strength with the distance. This is also a common assumption in analytical models and simulation tools. Nevertheless, our results confirm the findings of previous experimental campaigns, which demonstrate that this property is not true in practice. Moreover, the deviations from this predicted behavior seem to be exacerbated by a deployment at ground level. This also indicates the need for a denser deployment, which would allow coping with the poor quality of certain links. At a protocol level, opportunistic links are not uncommon and could be exploited.

Recommendation #3: The communication channel 26 must be preferable and frequency hopping is better.

Many studies on WSN do not consider the communication channel as a parameter, assuming the same quality on all transmission frequencies. In most field tests, designers evaluate their protocols on their testbed using only one channel. The results presented in Figure 10 and Figure 11 show that communication performance varies depending on the channel used by the nodes for communication. In the particular case of IEEE 802.15.4, our results indicate that communication on channel 26, which is not shared with other technologies, is probably a good idea. However, the WSN performance should be tested on several communication channels in order to find the one with the most reduced interference. Frequency hopping on several channels will lead to a better robustness.

Recommendation #4: At ground level, high density or redundant deployment will be preferable.

The results presented in this paper, and particularly those presented Section 5.1, show that the link quality is worse at ground level when compared to a more classical deployment at height. Thus, in a WSN with nodes deployed at ground level, special measures must be taken not only during the design of the communication protocols, but also during the network deployment. If we assume that a link is usable only when its PRR is greater than or equal to 90% (a good link in Figure 4), then only 70% of the links of 6 m length will be valid in a WSN with nodes deployed at the ground level, compared to 100% for a deployment at height (Figure 6b). During network deployment at ground level, a higher node density should therefore be preferred, in order to achieve a higher redundancy in the network and reduce the distance between the sensors, thus ensuring a certain reliability of the network. The results presented in this paper suggest that the sensors density will depend on particular properties of the deployment area. Indeed, Figure 13 shows that, with nodes deployed at ground level, the link quality is even worse when the sensors are deployed on a hill compared to a flat area. However, increasing the node density in the network will also increase the contention and the collision at the medium access control layer. To deal with this issue, one will have to choose appropriate MAC layer protocols.

Recommendation #5: Very small messages should be avoided.

In several WSN applications, the data captured by a node are encoded by just few bits, e.g., a temperature value or a vehicle detection information. It might be tempting to transmit these data directly to a sink, in small packets with a payload of just a few bytes. This study showed that the link quality is poor at ground level, no matter the packet size. However, the results presented in Figure 8 show that messages with a very small application payload are more exposed. While we do not completely understand in depth this phenomenon, aggregating multiple values in a single larger message will not only increase the packet reception probability, but also allow the implementation of error-correcting codes.

Recommendation #6: During the sensors deployment, the environment constraints must be taken into account.

The topography of the area where sensors are deployed, the networks and objects nearby, and the weather are some environment constraints that might have a negative impact on the radio link properties. In this work, we evaluated the impact of the topography of the environment by comparing the radio link properties considering a flat and a hill area. On the hill area, we did not measure the slope of the area. Nevertheless, Figure 13 shows a very poor radio link properties on the hill. Moreover, the results presented in Section 5.1.3 show that, in the same network, the link properties also depend on the location of the sensor. Thus, to guarantee a reliable communication, during the sensors deployment or during communication protocols design, the environment characteristics must be taken into account.

## 8. Conclusions

Thanks to their potential application, WSN with sensors deployed at ground level will play a bigger role in the future. we studied the properties of the radio link in a WSN with nodes deployed in such conditions. Our goal was not only to study the impact of the ground surface proximity on the radio link, but also to understand the impact of parameters such as the message size, the communication channel frequency or the topography of the environment on the quality of links between nodes deployed in such conditions.

For this, we conducted an extensive experimental campaign, comparing radio links at ground level with radio links in a network deployed above the ground. Our results indicate the feasibility of a WSN with nodes deployed at the ground level using off-the-shelf hardware. However, the link quality in this network is relatively poor compared with a deployment at height when considering the absolute values of the measured parameters (RSSI and PRR), the temporal behavior of the links, and the spatial or asymmetrical properties of links.

We evaluated the impact of several parameters, such as the message size, the communication channel frequency, and the topography of the deployment area in the case of the ground-level deployment. Our results show a deteriorated link quality when the message size is too small, when the communication channels shared by other technologies, e.g., WiFi, are used, or when sensors are deployed on a hill.

Finally, we summarized our results as recommendations for WSN designers who might be interested in deploying ground-level networks, for example in the case of large infrastructure monitoring. Some intriguing results obtained in our experiments, such as the poor PRR for packets with a very small application payload, or the poor link quality in the case of a hill deployment, open interesting research perspectives, which we plan to explore in the future. Source codes and experimental data are available online through the link https://github.com/rdomga/WirelessLinkCharacterization.

## Figures and Tables

**Table 1:** Link asymmetry for deployments at height and at ground level.

	Ground	Height
$AVG_{|PRR_{AB}-PRR_{BA}|}$	**0.0505**	0.04375
$MAX_{|PRR_{AB}-PRR_{BA}|}$	0.55	**0.6**
$AVG_{|{\bar{RSSI}}_{AB}-{\bar{RSSI}}_{BA}|}$	1.67	**2.67**
$MAX_{|{\bar{RSSI}}_{AB}-{\bar{RSSI}}_{BA}|}$	4.7	**14.001**
%Unidirectional	**8**	0

**Table 2:** Link asymmetry for different packet sizes.

	2 bytes	42 bytes	82 bytes
$AVG_{|PRR_{AB}-PRR_{BA}|}$	**0.2165**	0.05	0.0505
$MAX_{|PRR_{AB}-PRR_{BA}|}$	**0.9**	0.5	0.55
$AVG_{|{\bar{RSSI}}_{AB}-{\bar{RSSI}}_{BA}|}$	**1.94**	1.68	1.67
$MAX_{|{\bar{RSSI}}_{AB}-{\bar{RSSI}}_{BA}|}$	4.47	**5.0**	4.7
%Unidirectional	**18**	6	8

**Table 3:** Link asymmetry for different communication channels.

#Channel	11	14	18	22	26
$AVG_{|PRR_{AB}-PRR_{BA}|}$	0.09	0.01625	**0.0925**	0.078	0.0505
$MAX_{|PRR_{AB}-PRR_{BA}|}$	0.9	0.1	**1.0**	0.75	0.55
$AVG_{|{\bar{RSSI}}_{AB}-{\bar{RSSI}}_{BA}|}$	**2.35**	1.652	1.3	1.81	1.67
$MAX_{|{\bar{RSSI}}_{AB}-{\bar{RSSI}}_{BA}|}$	**11.67**	5.0	3.9	10.43	4.7
%Unidirectional	**40.83**	13.33	9.11	9	8

**Table 4:** Link asymmetry for flat and hill environments.

	Flat	Hill
$AVG_{|PRR_{AB}-PRR_{BA}|}$	0.0505	**0.1**
$MAX_{|PRR_{AB}-PRR_{BA}|}$	**0.55**	0.4
$AVG_{|{\bar{RSSI}}_{AB}-{\bar{RSSI}}_{BA}|}$	1.67	**4.3**
$MAX_{|{\bar{RSSI}}_{AB}-{\bar{RSSI}}_{BA}|}$	4.7	**14.75**
%Unidirectional	8	**45.83**

**Table 5:** Path-loss exponent and shadowing standard deviation for flat and hill deployment.

	α	σ
**Flat deployment**	3.56	6.08
**Hill deployment**	5.05	9.7
